# Decomposing contribution of age and non-age factors to rapid growth of lung cancer in Xuanwei over past 30 years

**DOI:** 10.1186/s12889-015-2482-y

**Published:** 2015-11-12

**Authors:** Yang Chen, Yize Xiao, Yongfang Yang, Jing Duan, Wen Xu

**Affiliations:** Department of Chronic disease, Yunnan center for disease control and prevention, Dongsi Street, Kunming, China

**Keywords:** Lung cancer, Mortality, Age, Non-age

## Abstract

**Background:**

From 1973 to 2005, the lung cancer mortality in Xuanwei had increased constantly. Effect analysis of age and non-age factors on lung cancer is important for local policy-making.

**Methods:**

Demographic and death data was collected and used. Factors of lung cancer were classified into age and non-age factors. The contribution of the two factors to lung cancer was evaluated by method of decomposing the differences of mortality rate.

**Results:**

For males, the non-age factors were the major contributor to growth of lung cancer mortality, and 78.46 % of all growth was attributed to non-age factors. For females, the non-age factors were the absolute contributor to growth of lung cancer in 1973–1992. From 1992 to 2005, the contribution proportion had reduced to 75.39 %.

**Conclusions:**

Aging was one of risk factors for lung cancer in Xuanwei, but not the main factor. It was supposed that multiple environmental risk factors were related with high growth of lung cancer in Xuanwei. Policy-making should focus on the non-age factors.

## Background

Xuanwei, a rural county, is located in the northeast of Yunnan Province,China. In 1973–1979, the age-adjusted mortality rate of lung cancer in Xuanwei (27.66 per 100 000 for men, 25.33 per 100 000 for women) was higher than that in Shanghai and Beijing (that is, 29.31 per 100 000 for men and10.81 per 100 000 for women, 14.81 per 100 000 for men and 11.31 per 100 000 for women in Shanghai and Beijing, respectively) ,and also higher than that in Kunming, the capital of Yunnan (13.86 per 100 000 for men, 3.95 per 100 000 for women) [1].

The accumulating evidence of epidemiologic, chemical and toxicologic studies has hypothesized that the etiology of lung cancer in Xuanwei is strongly associated with high exposure to the domestic emissions of “bituminous coal” (formerly known as “smoky coal”). A survey conducted in 1984 reported levels of submicron particles and carcinogenic polycyclic aromatic hydrocarbons(PAHs) compounds in emissions of “smoky coal” were higher than that in the emissions of “smokeless coal” [2]. 90 % of PAHs was adsorbed onto the submicron particles that was less than 2 μm [3]. Smaller particles showed stronger carcinogenicity [4]. Toxicological studies have shown that combustion products of the smoky coal are more tumorigenic and mutagenic than the products of smokeless coal [4, 5].

The ecology studies found that the commune-specific lung cancer mortality was highly related to the commune-specific percentage of homes using smoky coal before 1958 [2]. Epidemiological studies in Xuanwei have also found the lung cancer risk of smoky coal users was 6.05 times higher than that of smokeless coal users [6]. The retrospective cohort study showed that lung cancer alone accounted for about 40 % of all deaths before age 60 among individuals using smoky coal, and lifelong use of smoky coal compared with smokeless coal use was associated with a 36-fold increase of lung cancer mortality in men and 99-fold increase in women [7].

By the mid-1980s, more than 80 % of the residents had changed fire pits to stoves installing chimney or portable stoves under assist of the Chinese government. Studies showed that changing fire pits to stoves installing chimney, the mutagenic activity of indoor combustion production in Xuanwei has decreased [8]. The retrospective cohort studies indicate that either stoves with chimney or portable stoves was effective to reduce the indoor air pollution and the incidence risk of lung cancer in Xuanwei [9, 10].

But in 2004–2005, the age-adjusted mortality of lung cancer in Xuanwei was up to 83.28 per 100 000 for both sexes (20.24 per100 000 for China at the same time), and the annual growth rate in 1992–2005 had exceeded that of Yunnan and China (that is 5.74 % for Xuanwei, 1.30 % for Yunnan and 2.19 % for China) [11]. Age is a risk factor related closely with lung cancer. The elders are more prone to suffer from lung cancer. In this work, we supposed that high mortality rate of lung cancer in Xuanwei was caused by aging (age factor, AF) and other factors (non-age factors, NAF), and calculated accurately and quantitatively the contribution proportion of AF and NAF to the lung cancer in Xuanwei, to guide more effectively the local policy-making. NAF classified as all factors except AF, including socio-economic factors, environmental factors, medical and health services and behaviors of humans.

## Methods

### Data collection

The annual age- and sex-specific demographic data was provided and permitted to use by local bureau of Statistics affiliated Government, major responsibility of which was for the statistics of population. In China, the population census was carried out every ten years, and the sample survey of population was carried out every five years. The annual number of population was obtained based on the census and sample survey.

The data on death was provided and permitted to use by the Yunnan centers for disease control and prevention. Data in three periods, 1973–1975, 1990–1992 and 2004–2005, was collected as a part of the national retrospective sampling surveys of death cause. All people lived in Xuanwei was included in subjects of the surveys. The sample size was 2528237 in 1973–1975, 3566456 in1990–1992, and 2729497 in 2004–2005.

For a variety of reasons, the coverage of vital registration system in China was low. To understand the death cause of Chinese peoples, the national retrospective sampling surveys of death cause were conducted for three times, 1973–1975, 1990–1992 and 2004–2005, under the leadership of the Ministry of Health of the People Republic of China. Data was collected in a standard way by the trained investigators. First, the name lists of the dead were obtained from police departments, funeral parlour, medical institutions, and elder peoples in villages. Second, the investigators entered the home of the dead to obtain the information with a standard questionnaire concerning name, sex, birth date, death date, cause of death, and medical treatment. If the information of death was unknown for a little dead person, Verbal Autopsy was used. The Chinese Code for Death Causes and Population was used to categorize and code cause of death in 1973–1975 and 1990–1992, and the categories of the 10th revision of the International Classification of Diseases was used in 2004–2005. The definition of lung cancer has kept consistently in the three surveys.

We had complied with the Declaration of Helsinki Ethical Principles for Medical Research Involving Human Subjects (World Medical Association 1989). Informed consent was obtained from participants’ relatives, such as parents or son and daughters.

### Statistical analysis

Data was stratified by gender and 5-year age group. Mortality of lung cancer was expressed as deaths per 100,000 populations. The method of decomposing the differences of mortality rate was used to evaluate the contribution of AF and NAF to the lung cancer. The calculations can be performed according to the formulas expressed in the following steps [12].

Step 1 To estimate the contribution value

$$ \begin{array}{l} diff\\ {}=CD{R}_{(y1)}-CD{R}_{(y2)}\kern1.25em \\ {} = \Sigma {\mathrm{M}}_{\mathrm{i}\left(\mathrm{y}1\right)}*{\mathrm{C}}_{\mathrm{i}\left(\mathrm{y}1\right)}-\Sigma {\mathrm{M}}_{\mathrm{i}\left(\mathrm{y}2\right)}*{\mathrm{C}}_{\mathrm{i}\left(\mathrm{y}2\right)}\\ {}=\Sigma \left[{\mathrm{C}}_{\mathrm{i}\left(\mathrm{y}1\right)}-{\mathrm{C}}_{\mathrm{i}\left(\mathrm{y}2\right)}\right]\ast \frac{\left[{\mathrm{M}}_{\mathrm{i}\left(\mathrm{y}1\right)}+{\mathrm{M}}_{\mathrm{i}\left(\mathrm{y}2\right)}\right]}{2}+\Sigma \left[{\mathrm{M}}_{\mathrm{i}\left(\mathrm{y}1\right)}-{\mathrm{M}}_{\mathrm{i}\left(\mathrm{y}2\right)}\right]*\frac{\left[{\mathrm{C}}_{\mathrm{i}\left(\mathrm{y}1\right)}+{\mathrm{C}}_{\mathrm{i}\left(\mathrm{y}2\right)}\right]}{2}\end{array} $$

Diff = difference of total mortality

CDR(y1) = total mortality in a population in a period

CDR(y2) = total mortality in a population in another period

Mi = the age specific mortality rates

Ci = the age specific proportion of population.

The contribution value of AF=

$$ \Sigma \left[{\mathrm{C}}_{\mathrm{i}\left(\mathrm{y}1\right)}-{\mathrm{C}}_{\mathrm{i}\left(\mathrm{y}2\right)}\right]\ast \frac{\left[{\mathrm{M}}_{\mathrm{i}\left(\mathrm{y}1\right)}+{\mathrm{M}}_{\mathrm{i}\left(\mathrm{y}2\right)}\right]}{2} $$

The value of NAF=

$$ \Sigma \left[{\mathrm{M}}_{\mathrm{i}\left(\mathrm{y}1\right)}-{\mathrm{M}}_{\mathrm{i}\left(\mathrm{y}2\right)}\right]*\frac{\left[{\mathrm{C}}_{\mathrm{i}\left(\mathrm{y}1\right)}+{\mathrm{C}}_{\mathrm{i}\left(\mathrm{y}2\right)}\right]}{2} $$

Step 2 To estimate the contribution proportion

The contribution proportion of AF=

$$ \Sigma \left[{\mathrm{C}}_{\mathrm{i}\left(\mathrm{y}1\right)}-{\mathrm{C}}_{\mathrm{i}\left(\mathrm{y}2\right)}\right]\ast \frac{\left[{\mathrm{M}}_{\mathrm{i}\left(\mathrm{y}1\right)}+{\mathrm{M}}_{\mathrm{i}\left(\mathrm{y}2\right)}\right]}{2}/ diff*100 $$

The contribution proportion of NAF=

$$ \Sigma \left[{\mathrm{M}}_{\mathrm{i}\left(\mathrm{y}1\right)}-{\mathrm{M}}_{\mathrm{i}\left(\mathrm{y}2\right)}\right]*\frac{\left[{\mathrm{C}}_{\mathrm{i}\left(\mathrm{y}1\right)}+{\mathrm{C}}_{\mathrm{i}\left(\mathrm{y}2\right)}\right]}{2}/ diff*100 $$

Data analysis was performed in Excel 2010.

## Results

**Fig. 1:**
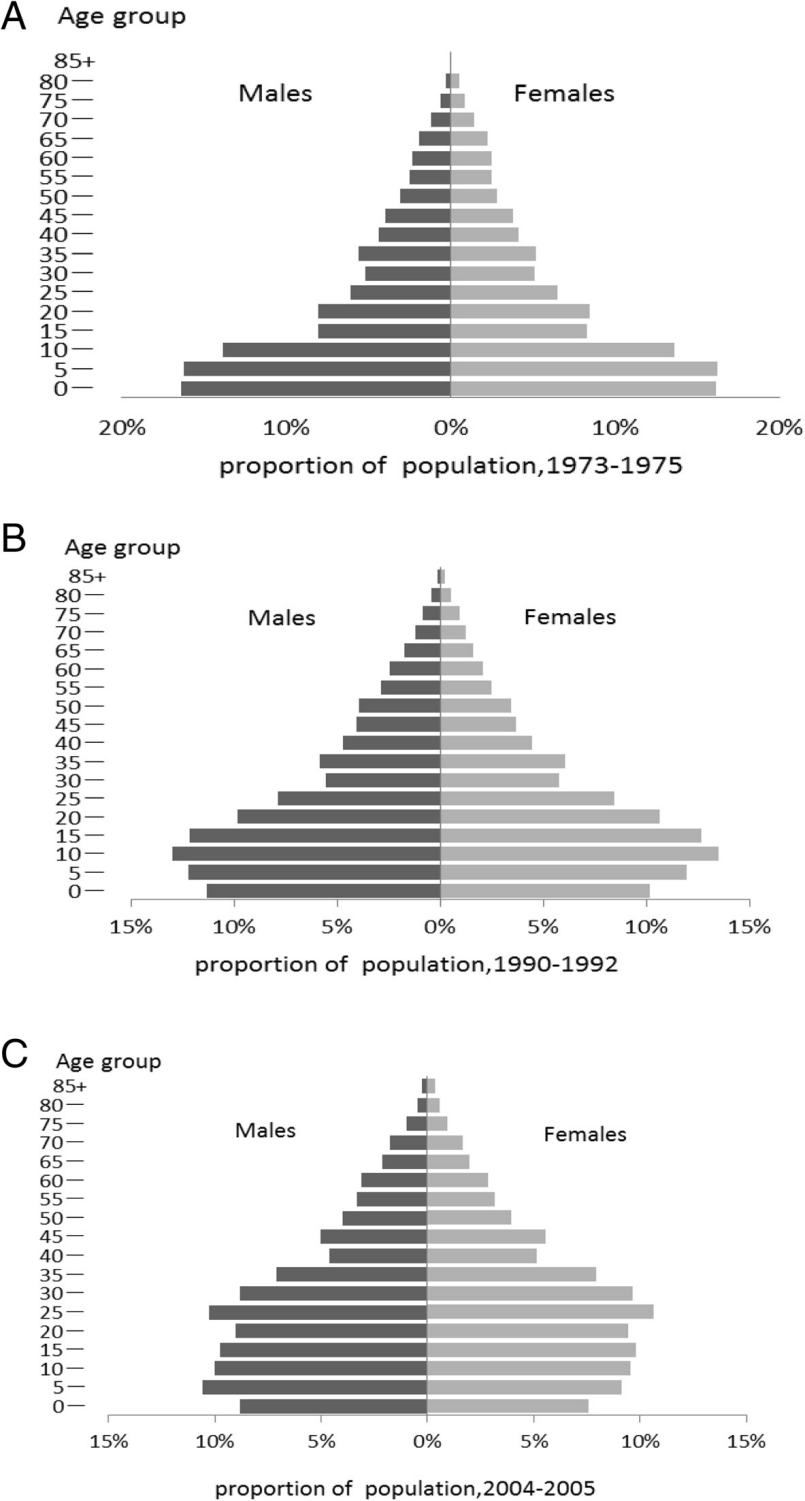
The people pyramid of Xuan Wei in three periods. **a** composition of the population(horizontal axis) and age groups(vertical axis) in 1973–1975. **b** composition of the population(horizontal axis) and age groups(vertical axis) in 1990–1992. **c** composition of the population (horizontal axis) and age groups(vertical axis) in 2004–2005

Table 1 presents the proportion of population by sex, age groups in Xuanwei in 1973–1975, 1990–1992, and 2004–2005. Figure 1 illustrates that the population pyramid in Xuanwei in the three periods. It highlights the growing proportion of older population in parallel with a decreasing proportion of younger population. The triangular young population pyramid of 1970s’ was already replaced with an aged structure in 2000s’.

**Table 1:** The proportion of population by age and sex group in Xuan Wei in three periods (%)

Age	Males			Females			Total		
	1970s’	1990s’	2000s’	1970s’	1990s’	2000s’	1970s’	1990s’	2000s’
0-	16.44	11.33	8.84	16.10	10.20	7.60	16.28	10.79	8.25
5-	16.23	12.19	10.58	16.21	11.94	9.15	16.22	12.07	9.91
10-	13.83	12.96	9.99	13.59	13.49	9.54	13.71	13.21	9.78
15-	8.09	12.14	9.77	8.28	12.65	9.81	8.18	12.39	9.79
20-	8.09	9.82	9.02	8.43	10.64	9.43	8.25	10.21	9.21
25-	6.09	7.84	10.27	6.46	8.45	10.64	6.27	8.13	10.44
30-	5.17	5.53	8.83	5.07	5.80	9.64	5.12	5.65	9.21
35-	5.61	5.84	7.09	5.21	6.10	7.94	5.42	5.96	7.49
40-	4.42	4.72	4.64	4.10	4.47	5.15	4.27	4.60	4.88
45-	4.00	4.08	5.02	3.79	3.68	5.57	3.90	3.89	5.28
50-	3.08	3.94	3.99	2.80	3.46	3.97	2.94	3.71	3.98
55-	2.54	2.88	3.30	2.46	2.48	3.18	2.50	2.69	3.24
60-	2.35	2.45	3.09	2.48	2.11	2.87	2.41	2.29	2.99
65-	1.92	1.73	2.14	2.21	1.62	1.96	2.06	1.68	2.06
70-	1.21	1.18	1.76	1.45	1.25	1.64	1.33	1.21	1.71
75-	0.64	0.85	0.96	0.83	0.94	0.96	0.73	0.89	0.96
80-	0.29	0.40	0.47	0.52	0.53	0.57	0.40	0.46	0.52
85+	0.00	0.13	0.24	0.00	0.22	0.37	0.00	0.17	0.30
total	100.00	100.00	100.00	100.00	100.00	100.00	100.00	100.00	100.00

Table 2 shows mortality rate of lung cancer by 5-year age group and sex in Xuanwei in three periods. Lung cancer mortality rate showed a clear upward trend. The mean annual death rate of lung cancer for all age groups is 23.14 per 100 000 population (24.81 for men and 21.35 for women) in 1973–1975, whereas 34.88 per 100 000 population (37.92 for men and 31.56 for women) in 1990–1992 and 91.30 per 100 000 population (98.24 for men and 83.46 for women) in 2004–2005. The male/female ration in three periods was 1.16:1.00, 1.20:1.00 and 1.18:1.00 respectively.

**Table 2:** The sex- and age- specific mortality of lung cancer in Xuan Wei in three periods (per 100 000)

Age	Males			Females			Total		
	1970s	1990s	2000s	1970s	1990s	2000s	1970s	1990s	2000s
0-	0.00	0.00	0.00	0.00	0.58	0.00	0.00	0.26	0.00
5-	0.00	0.00	0.00	0.00	0.49	0.00	0.00	0.23	0.00
10-	0.00	1.24	0.00	0.00	0.43	0.00	0.00	0.85	0.00
15-	0.95	1.77	0.71	0.00	0.93	1.59	0.48	1.36	1.12
20-	0.00	3.83	3.83	0.97	3.86	0.83	0.48	3.84	2.39
25-	2.51	7.54	6.05	2.53	6.25	2.20	2.52	6.90	4.21
30-	7.41	18.47	30.51	4.84	14.17	11.33	6.18	16.36	21.08
35-	12.29	24.82	60.40	10.99	17.32	43.23	11.68	21.16	51.85
40-	32.92	48.97	146.03	43.89	35.44	87.86	38.01	42.69	117.94
45-	91.89	77.72	147.28	69.06	87.56	100.77	81.16	82.17	124.91
50-	104.43	126.84	256.16	131.45	140.75	216.16	116.85	133.04	237.42
55-	220.09	216.02	318.69	189.51	251.01	319.36	205.55	231.43	319.00
60-	205.29	322.25	395.17	148.41	244.78	467.19	177.02	288.10	427.66
65-	135.60	208.18	661.82	88.82	173.80	659.74	111.34	192.30	660.89
70-	120.24	264.76	631.19	101.51	206.62	554.96	110.34	236.10	596.69
75-	71.79	253.63	1003.19	49.24	143.81	609.41	59.42	198.34	818.66
80-	79.22	148.51	1195.39	0.00	99.60	912.19	29.55	121.63	1048.08
85+	0.00	42.61	1004.16	0.00	54.22	824.18	0.00	49.70	900.52
total	24.81	37.92	98.24	21.35	31.56	83.46	23.14	34.88	91.30

**Fig. 2:**
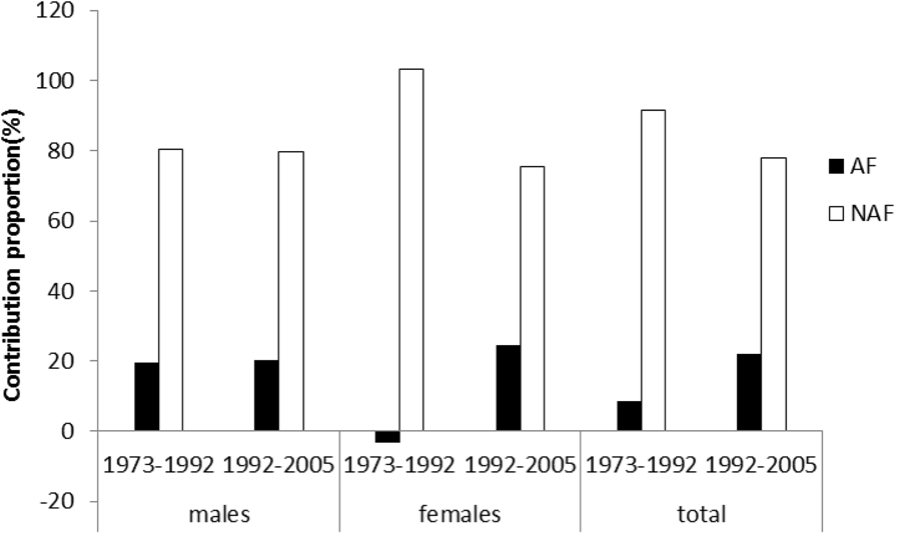
Contribution proportion of AF and NAF to lung cancer in Xuan Wei in three periods

Table 3 and Fig. 2 shows the contribution proportion of AF and NAF to the growth of lung cancer mortality rate in latest 30 years. For males, approximately 80 % of the growth was attributed to the NAF in 1973–2005 (80.56 % in 1973–1992, 79.87 % in 1992–2005, and 78.46 % in 1973–2005). For females, the NAF was the absolute contributor (103.20 %) in 1973–1992. Meanwhile, the AF was a protective factor just due to the reduction of 60–70 years females in 1992. The contribution proportion of AF increased to 24.61 %, meanwhile the contribution proportion of the NAF reduced to 75.39 % in 1992–2005. For all people, the NAF was the major contributor all the time (91.36 % in 1973–1992,77.91 % in 1992–2005, and 80.76 % in 1973–2005).

**Table 3:** Contribution proportion of AF and NAF to growth of lung cancer mortality in Xuanwei during three periods

Contribution	1973–1992			1992–2005			1973–2005		
	AF	NAF	Total	AF	NAF	Total	AF	NAF	Total
Males Contribution value (difference of mortality)	2.55	10.56	13.11	12.14	48.17	60.31	15.81	57.61	73.42
Contribution proportion(%)	19.44	80.56	100	20.13	79.87	100	21.54	78.46	100
Females Contribution value (difference of mortality)	−0.33	10.53	10.2	12.77	39.13	51.9	10.38	51.73	62.11
Contribution proportion(%)	−3.2	103.2	100	24.61	75.39	100	16.71	83.29	100
Both sexes Contribution value (difference of mortality)	1.01	10.73	11.74	12.46	43.96	56.42	13.11	55.04	68.15
Contribution proportion(%)	8.64	91.36	100	22.09	77.91	100	19.24	80.76	100

## Discussion

Mortality and incidence of cancers in China have been increasing over the past 30 years, mainly due to the AF. Meanwhile, lung cancer have become the first cause among all cancers caused death, mainly due to the NAF [13]. From 1973 to 2005, the lung cancer mortality in Xuanwei had increased constantly, and NAF was the main cause of the increasing trend. Compared with a proportion of 63 % for China at the same time [12], the influence of NAF on lung cancer in XuanWei was more serious.

The high mortality rate of lung cancer in Xuanwei was once attributed to high PAH concentration from bituminous coal burned indoor. In recent decades, most residents of Xuanwei have changed fire pits to stoves with chimney or portable stoves, but which did not eliminate the effect of bituminous coal. In 2009, Lin and colleagues collected samples of indoor and outdoor air from 90 different family residences in Laibin and Reshui communes in Xuanwei,and determined the concentration of benzo(a)pyrene(Bap). The result showed that the concentration of benzo(a)pyrene of indoor air in both towns seriously exceeded the national indoor air quality standard. The excessive multiples were 132 and 64 respectively. For outdoor air in the two towns, benzo(a)pyrene was beyond the national environmental air quality standard limits [14].

Tian and colleagues collected township-based lung cancer mortality of 4 counties (Xuanwei, Fu Yuan, Zhan Yi, and Qi Lin) and coal geology data, and found Lung cancer rates tend to be higher in place where C1 coal, the uppermost coal seam of Late Permian, is produced. Meanwhile Nano-sized quartz has also been identified in soot emissions from C1 coal combustion [15]. A geological study reported that the coal collected from Laibin district, an area with high lung cancer mortality rate in Xuanwei, has unusual high concentration of quartz (13.5 wt%), of which 35–55 % occurs as <10 μm, and silica-volatile interaction is correlated with lung cancer mortality in Xuanwei (*p* = 0.661, *p* = 0.091). Further, it supposed that a likely way for women exposed fine-grained silica was dumping bottom ash of coal combustion, which contained abundant of fine-grained silica [16]. A population-based case–control study of lung cancer in Xuanwei found that the carcinogenic risk of coal subtype was varied. Estimates were highest for coal from the Laibin (OR = 24.8) and Longtan (OR = 11.6) [17]. An experiment in vitro cytotoxicity showed that the bottom ash products of coal combustion from Laibin enrich in free silica, and compared to the control and blank group, the viability of bronchial epithelial cells BEAS-2B in experimental group exposed to bottom ash of coal combustion from Laibin is significantly lower. The oxidative damage to BEAS-2B of bottom ash of coal from Laibin is more serious [18].

The smoking prevalence in residents aged 30–79 in 5 counties (Xuanwei, Fu Yuan, Shi Zong, Qi Lin and Luo Ping) was 85.05 % of all men and 1.37 % of all women [19]. Strong evidence of a link between smoking and lung cancer has existed since 1950 [20]. The global deaths of Trachea, Bronchus, Lung cancers attributable to tobacco was 71 % for 30 years and over in 2004 [21]. In Xuanwei, smoking was not suggested as a main risk factor of lung cancer in early studies. In 2008, Lan et al. found a relatively weak effect of smoking with lung cancer risk, and proposed that combined exposure to tobacco smoke and very high indoor air pollution levels tended to attenuate the observed effect of smoking [17]. But in 2010, Lee et al. estimated the effect of smoking on lung cancer after stove improvement in Xuanwei, and found that the effect of smoking on lung cancer have become considerably stronger after chimney installation and consequent reduction of indoor coal smoke exposure [22].

The females are the main victim of passive smoking. A case–control study conducted in Beijing in non-smoking women showed that adenocarcinoma of lung was more possibly induced by environmental tobacco smoke (ETS, OR = 2.32, *P* < 0.05) . However, the association between squamous cell cancer and ETS was not quite as strong as that for adenocarcinoma (OR = 1.04, *P* < 0.05) [23]. Whereas, a Meta-analysis of risk on lung cancer in non-smoking Chinese females demonstrated that the effect on cancer of exposure to ETS was uncertain [24]. Liu and colleagues reported that passive smoking was not associated with lung cancer in women in Xuanwei [25]. Hou analyzed pathological types of 141 patient with lung cancer lived in Xuanwei and discovered that the majority of lung cancer was adenocarcinomas of lung. It was unknown that whether the phenomenon was associated with ETS [26].

Application of new screening method in clinic may found more patients with lung cancer. However, which cannot explain the growth of lung cancer. Xuanwei is a rural county in the northeast of Yunnan Province which economy condition was among the last five Provinces’ of China. So, if there are not special factors, the mortality of lung cancer in Xuanwei should lower than that in Beijing or Shanghai, which economy status or development level are more better. In fact,the mortality of lung cancer in Beijing and shanghai are 40.09 per 100 000 and 51.51 per 100 000 respectively in 2004–2005, that was more lower than that of Xuanwei (83.28 per 100 000) [27]. Furthermore, the detection carried out in Xuanwei may also found actively more patients with lung cancer. To the best of our knowledge, there are not any detection project in Xuanwei at the survey periods. From 2007, under assist of the Chinese and Yunnan government,some detection projects were carried out in part of villages in Xuanwei,but that cannot affect our results.

## Conclusions

In summary, we supported that age is one of the factors caused the high growth of lung cancer in XuanWei, but is not the main factor. Policy-making should focus on the NAF.It is supposed that multiple environmental risk factors caused jointly the high growth of lung cancer. Except for PAH from bituminous coal combustion, Nano-sized crystalline silica in the air or bottom ash maybe plays an important role. Smoking is popular in the males in Xuanwei, so the smoking control for men is also important.
